# A multi-disciplinary model of survivorship care following definitive chemoradiation for anal cancer

**DOI:** 10.1186/s12885-019-6053-y

**Published:** 2019-09-11

**Authors:** Marissa B. Savoie, Angela Laffan, Cristina Brickman, Bevin Daniels, Anna Levin, Tami Rowen, James Smith, Erin L. Van Blarigan, Thomas A. Hope, J. Michael Berry-Lawhorn, Mekhail Anwar, Katherine Van Loon

**Affiliations:** 1 School of Medicine, University of California, San Francisco, USA; 2 Helen Diller Family Comprehensive Cancer Center, University of California, San Francisco, USA; 3 Department of Medicine, Division of Infectious Diseases, University of California, San Francisco, USA; 4 Department of Physical Therapy, University of California, San Francisco, USA; 5 Department of Psycho-Oncology, University of California, San Francisco, USA; 6 Department of Obstetrics, Gynecology and Reproductive Sciences, University of California, San Francisco, USA; 7 Department of Urology, University of California, San Francisco, USA; 8 Department of Epidemiology and Biostatistics, University of California, San Francisco, USA; 9 Department of Radiology and Biomedical Imaging, University of California, San Francisco, USA; 10 Department of Medicine, Division of Hematology/Oncology, University of California, San Francisco, USA; 11 Department of Radiation Oncology, University of California, San Francisco, USA

**Keywords:** Anal cancer, Survivorship, Toxicity, Surveillance

## Abstract

Following definitive chemoradiation for anal squamous cell carcinoma (ASCC), patients face a variety of chronic issues including: bowel dysfunction, accelerated bone loss, sexual dysfunction, and psychosocial distress. The increasing incidence of this disease, high cure rates, and significant long-term sequelae warrant increased focus on optimal survivorship care following definitive chemoradiation. In order to establish our survivorship care model for ASCC patients, a multi-disciplinary team of experts performed a comprehensive literature review and summarized best practices for the multi-disciplinary management of this unique patient population. We reviewed principle domains of our survivorship approach: (1) management of chronic toxicities; (2) sexual health; (3) HIV management in affected patients; (4) psychosocial wellbeing; and (5) surveillance for disease recurrence and survivorship care delivery. We provide recommendations for the optimization of survivorship care for ASCC patients can through a multi-disciplinary approach that supports physical and psychological wellness.

## Background

Anal squamous cell carcinoma (ASCC) is a rare cancer, with only 8580 cases diagnosed in the United States annually. However, the incidence has steadily increased at a rate of 2.2% per year over the last 10 years [1]. ASCC is mediated by the human papilloma virus (HPV) in 85–95% of cases [2–4]. Accordingly, risk factors for ASCC largely overlap with risk factors for HPV infection, including: human immunodeficiency virus (HIV) infection, solid-organ transplantation, multiple sexual partners, anal-receptive intercourse, anal warts, history of cervical, vulvar, or vaginal carcinoma, chronic immune suppression, and cigarette smoking [5, 6]. However, this disease also occurs in the absence of immunocompromise and numerous sexual partners. While ASCC is rare, the prevalence of anal HPV in women is comparable to that of cervical HPV in women [7], and HPV transmission can occur during vaginal intercourse due to contamination of the entire perineal area. In fact, HIV-negative females without identified high-risk behaviors are over-represented in the rising incidence of this disease [8, 9].

Intensive radiation with concurrent 5-flurouracil and mitomycin is the standard treatment for locoregional ASCC [10–12]. The advent of more advanced radiotherapy techniques, such as intensity-modulated radiation therapy (IMRT), has allowed for more focused radiation and is associated with lower rates of chronic toxicities [13]. Given the enhanced ability to tailor radiation treatment with IMRT, there is an increased emphasis on understanding long-term side effects so that these can be taken into account during radiation planning. However late toxicity data is sparse, reflecting the paucity of long-term data collection in therapeutic trials [14, 15].

Cure rates exceed 80% for the 90% for patients diagnosed with locoregional disease [1], and the majority of patients diagnosed with ASCC will become long-term survivors. However, even after being rendered disease-free, patients face myriad treatment-related sequelae over subsequent decades. Specifically, ASCC survivors report a high prevalence of chronic toxicities including: sexual dysfunction, bowel dysfunction, accelerated bone loss, cognitive changes, and fatigue [16]. Additionally, care for ASCC survivors is wrought with issues related to social isolation due to perceptions of stigma [17].

The University of California, San Francisco (UCSF) is home to the Anal Neoplasia Clinic, which was founded in 1991 to address increased rates of anal dysplasia, a precursor of ASCC, observed in people living with HIV (PLWH) [18]. This clinic serves to diagnose anal dysplasia and also to monitor for ASCC recurrence in survivors. Patients from the San Francisco and Oakland region are more likely to have in-situ tumors than patients from other areas of California; this has been attributed in part to the accessibility of successful screening efforts [19]. Given higher rates of HIV infection in the San Francisco Bay Area and increased rates of curable disease compared to national populations, our institution cares for a high volume of ASCC survivors.

In order to establish our survivorship care model for ASCC patients, a multi-disciplinary team of experts in the Gastrointestinal Oncology Survivorship practice at our institution performed a comprehensive literature review of the survivorship issues and summarized best practices for the management of this unique patient population. Herein, we describe our multi-disciplinary approach to caring for patients with ASCC following curative treatment. The principle components of this approach include: (1) management of chronic toxicities; (2) sexual health; (3) HIV management in affected patients; (4) psychosocial wellbeing; and (5) surveillance for disease recurrence and survivorship care delivery.

## Management of chronic toxicities

### Bowel dysfunction

CRT for ASCC involves a variable amount of radiation delivered to the rectum, large and small intestines. While guidelines exist governing the maximum recommended dose for bowel, tumor stage, bulk, proximity to bowel, and variations in patient anatomy result in significant heterogeneity in bowel exposure to radiation. Not unexpectedly, patients commonly experience bowel dysfunction following CRT with variable onset, degree and duration, ranging from months to years. Long-term toxicity data from clinical trials of chemotherapy with 3D radiation and IMRT report moderate or higher gastrointestinal toxicity rates of approximately 10% [11] and 8–10% [20, 21] respectively (Table 1).

**Table 1:** Rates of late (> 90 days after last chemoradiation therapy) gastrointestinal toxicity among anal cancer patients

Study	Grading system	Grade 1	Grade 2+	Grade 3+
Mitra et al. [21] 2017 (IMRT)	CTCAE v4.0 [22]	37%	10%	2%
Mitchell et al. [20] 2014 (IMRT)	CTCAE v4.0 [22]	Not given	8%	3%
Tomaszewski et al. [23] 2012	CTCAE v4.0 [22]	Not given	Not given	3%
Gunderson et al. [12] 2012	RTOG and EORTC toxicity criteria [24]	Not given	Not given	2%
Ajani et al. [11] 2008	RTOG and EORTC toxicity criteria [24]	16%	8%	3%

*IMRT* intensity-modulated radiation therapy, *CTCAE* common terminology criteria for adverse events, *RTOG* Radiation Therapy Oncology Group, *EORTC* European Organisation for Research and Treatment of Cancer

Among cancer patients of various tumor types, approximately half of patients who receive treatment with pelvic radiation report their quality of life following treatment is compromised by gastrointestinal symptoms > 3 months after completion of treatment [25–27]. These patients report that fecal urgency, incontinence, and tenesmus impede quality of life by causing emotional distress and disruptions to social function [26]. One study demonstrated that 29% of ASCC survivors reported changes in bowel patterns [16], and another demonstrated that 26% of patients report the effect of diarrhea on quality of life as “quite a bit or higher” [28].

Diarrhea is the most cited gastrointestinal concern that impacts quality of life following treatment for ASCC [21, 25, 29–35]. Diarrhea can alternate with constipation and be accompanied by abdominal cramping, rectal bleeding, or pain [25, 30, 34]. Management of diarrhea can range from dietary modifications and use anti-diarrheal medications to procedures performed by specialists which target the site of tissue injury [36]. Fecal urgency is often noted; however, this is likely underreported in the literature as current toxicity scales do not allow for categorization of urgency or tenesmus as a complaint independent from proctitis [22, 24]. Several studies of late radiation toxicity in ASCC patients indicate that severity of bowel dysfunction is stable in the years following CRT [31, 37], indicating the potential for persistent, rather than resolving, late bowel toxicities.

**Fig. 1:**
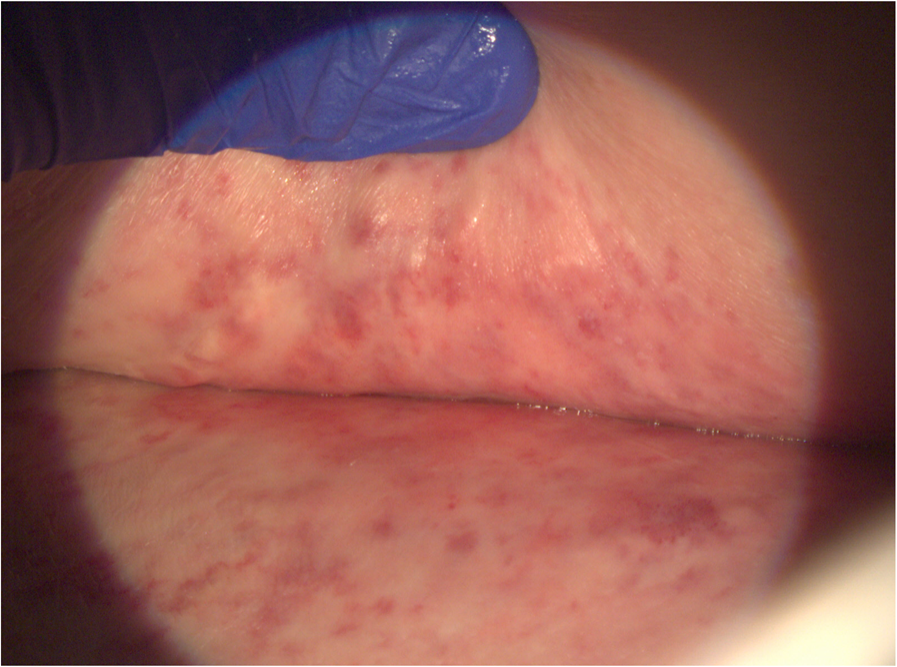
Perianal telangiectasias. Perianal telangiectasias seen on the skin after radiation therapy for anal squamous cell carcinoma

In a patient who presents with diarrhea and hematochezia after CRT for ASCC, colonoscopy or high-resolution anoscopy (HRA) and/or CT or MRI may be warranted to differentiate chronic radiation enteritis or proctitis from other causes. In the setting of chronic radiation enteritis, biopsies demonstrate increased friability and telangiectasias, consistent with ischemic injury, and CT/MRI demonstrate bowel inflammation with hyperenhancement and bowel thickening [25]. The presence of peri-anal telangiectasias on external exam (Fig. 1) may signal providers to consider similar internal pathology. Mainstays of managing chronic radiation enteritis include antidiarrheal medicines and adherence to a lactose-free, low-fiber, and low-fat diet [38–40]; steroids and pain medication can be considered with initial presentation. Surgery to mitigate fibrosis, adhesions, or fistulae secondary to radiation enteritis is a last recourse, as short bowel syndrome is a potential complication [41–43].

While radiation enteritis involves damage to the intestines, late proctitis is a manifestation of ischemic radiation injury to the rectum and may present as diarrhea, bleeding, tenesmus, and/or urgency years after radiation exposure [44, 45]. After infectious etiology has been excluded by history and/or cultures, a rectal biopsy should be considered; the findings of friability and telangiectasias are nonspecific for radiation proctitis. Sucralfate enemas have been shown to decrease rectal bleeding [46–50]. Directed interventions such as argon plasma coagulation and formalin treatment to the rectal site of injury can be utilized to quell bleeding although these interventions carry risks for fistula formation [51–61]. Hyperbaric oxygen treatment has been shown improve radiation proctitis symptoms and increase probability of healing [62, 63]. This was shown among patients who had radiation proctitis refractory to other treatments for > 3 months, suggesting that hyperbaric oxygen should be reserved as an intervention for refractory symptoms [63].

Given the variable latency of late radiation bowel dysfunction, a new presentation of inflammatory bowel disease, infectious gastroenteritis, or acute proctitis secondary to sexually transmitted infection should also be considered. Other causes of bowel dysfunction related to pelvic radiation include bacterial overgrowth and fibrosis. If bacterial overgrowth is suspected due to history of chronic malabsorptive diarrhea in the absence of other identified causes, carbohydrate breath test may aid in diagnosis but is limited by low sensitivity [64]. Potential interventions for bacterial overgrowth include antibiotics, with rifaximin as the preferred initial agent [64]. Like chronic radiation enteritis, generalized gastrointestinal radiation toxicities can be managed with trial(s) of lactose-free and/or a BRAT diet [25, 27, 65].

### Accelerated bone degeneration

As a key component to curative treatment for ASCC, external beam radiotherapy targets the primary tumor and involved nodes, as well as areas at high risk for subclinical or microscopic disease. Innovations over the last two decades in radiotherapy delivery have moved from 2D or 3D techniques to highly conformal IMRT. This allows personalized delivery with maximum dosing to key areas (gross or visible tumor) and elective dosing to at-risk areas. Notably, this customized sculpting keeps high-dose radiation away from critical bone regions [66], such as the femoral heads, with the tradeoff of a larger area of bone exposed to lower dose radiation.

**Fig. 2:**
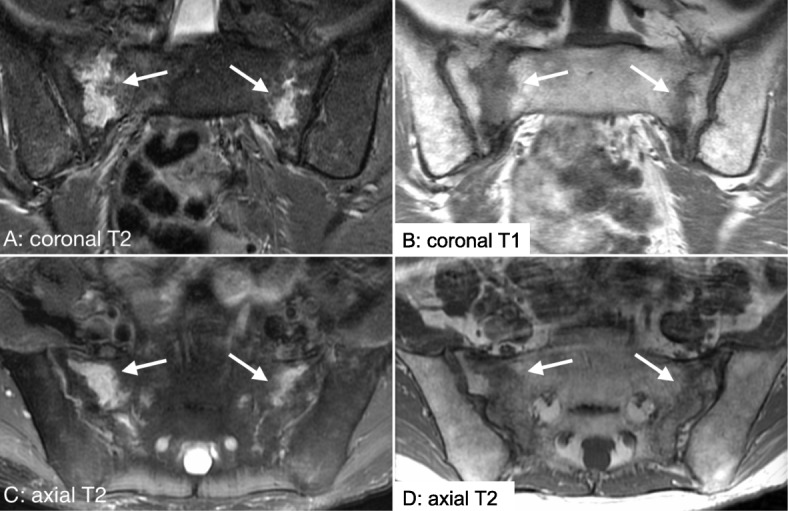
Pelvic insufficiency fracture. Coronal T1 (Image **b**), coronal T2 (Image **a**) and axial T2 (Image **c, d**) weighted images demonstrating high T2 signal bilaterally along the sacroiliac joints consistent with bilateral sacral insufficiency fractures

Pelvic insufficiency fractures (PIFs) are the most commonly discussed chronic bone toxicity of pelvic radiation [67, 68]; however, osteoradionecrosis and osteomyelitis are rarer, late complications of pelvic radiation [69, 70]. PIFs following pelvic radiation most often occur in the sacrum, pubic symphysis, or pubic rami [67, 71, 72]. While the limited studies evaluating the late effects of CRT in ASCC patients have not found adequate evidence to determine a consensus around risk of PIFs for these patients, one study noted a 9.3% incidence rate of PIFs in ASCC patients treated with IMRT with a median follow-up interval of 3.1 years (Table 2) [72]. Higher radiation dose, post-menopausal status in females, low BMI, and prior osteoporosis have been observed as risk factors for PIF in patients who have received pelvic radiation [72, 73, 75, 77, 83]. Patients with treated HIV have an elevated risk for decreased bone mineral density in the hip and lumbar spine region at baseline and they may be at a heightened risk for PIF [85]. Figure 2 provides an example of bilateral sacral insufficiency fractures in a patient who received pelvic radiation for ASCC.

**Table 2:** Summary of studies measuring incidence of pelvic insufficiency fractures (PIFs) among cancer patients receiving pelvic radiation therapy

Study	Era	No. patients	Median time to fracture/total follow-up	Median total dose (Gray)	Disease site	Study Incidence^a^	5-year actuarial incidence^b^
Bazire et al. [72] 2017	2007–2014	341	11 mo./38 mo.	50.3	52% cervical 32% endometrial 16% anal	4.4% Radiographic (R)	Not given
						3.2% Symptomatic (S)	
Shih et al. [73] 2013	2000–2008 37% IMRT	222	12 mo./47 mo.	50.4	65% endometrial 35% cervical	5.0% (R)	5.1% (R)
						3.2% (S)	
Uezono et al. [74] 2013	2003–2009	99	14 mo./21 mo.	50.4	cervical	33% (R)	63% (R)
						20% (S)	
Kim et al. [75] 2012	1998–2007	492	46 mo./42 mo.	50.4	rectal	7.1% sacral fracture (R)	Not given
Tokumaru et al. [76] 2012	2004–2007	59	not given/24 mo.	49	cervical	36%(R)	Not given
						15% (S)	
Schmeler et al. [77] 2010	2001–2006 3% IMRT	300	14 mo./21 mo.	45	cervical	9.7% (R)	Not given
						4.3% (S)	
Herman et al. [78] 2009	1989–2004	562	17 mo./49 mo.	45	rectal	2.7% sacral fracture (R)	Not given
						1.2% sacral fracture (S)	
Oh et al. [79] 2008	1998–2005	557	13 mo./30 mo.	45	cervical	15%(R)	20% (R)
						8.6% (S)	11% (S)
Kwon et al. [80] 2008	1998–2005	510	17 mo./14 mo.	50.4	cervical	20% (R)	45% (R)
						8.4% (S)	
Ikushima et al. [81] 2006	1993–2004	158	6 mo./43 mo.	45	96% cervical 4% endometrial	11% (S)	13% (S)
Baxter et al. [82] 2005	1986–1999	399 women age 65+	not given/47 mo.	Not given	anal	14% (unclear R/S)	14% (unclear R/S)
Ogino et al. [83] 2003	1983–1998	335 post-menopausal	8 mo./39 mo.	49.4	cervical	17% (R)	18% (S)
						14% (S)	
Tai et al. [84] 2000	1991–1995	336	11 mo./29 mo.	Not given	endometrial vaginal	4.8% (S)	2.1% (S)

^a^Incidence calculated over variable study period

^b^5-year actuarial incidence calculated with Kaplan Meier analysis

PIFs are not uncommon among ASCC patients following CRT; however, the complications from these fractures are usually self-limited. The clinical presentation of PIF rarely requires hospitalization or extensive intervention beyond pain management [78, 79, 81, 83, 86, 87]. Considering these findings, screening for osteopenia or osteoporosis should be performed following completion of CRT with a dual-energy x-ray absorptiometry (DEXA) scan. Supplementation of vitamin D and calcium, oral bisphosphonates, or anabolic agents may be warranted, as recommended by age-appropriate preventative care recommendations and the National Comprehensive Cancer Network (NCCN) Guidelines for Oncology Survivorship [88].

### Pelvic floor dysfunction

Radiation treatment to the pelvis and anus can affect pelvic floor muscles, fascia, and nerves, resulting in pelvic floor dysfunction. Specifically, significant pelvic floor muscle weakness is common after pelvic radiation [89], and myofascial trigger points and increased muscle tension can cause pain. Research on pelvic floor physical therapy after cancer is largely derived from gynecologic and colorectal cancer patients and consistently demonstrates improvements in pelvic floor function [90, 91]. Extrapolating from existing literature, physical therapy directed towards the pelvic floor can play an important role in recovery and help improve quality of life after radiation treatment, particularly for survivors who present with fecal and/or urinary incontinence, dyspareunia, or sexual dysfunction.

Impairments in pelvic floor muscle strength, coordination and tone caused by radiation can be successfully treated with neuromuscular re-education, biofeedback, and electrical stimulation. Internal and/or external manual treatments, such as myofascial release, connective tissue manipulation, and joint mobilization to the pelvis, spine and hips, may be used to treat pelvic pain. Vaginal and/or anal dilation therapy is commonly incorporated as a component of pelvic rehabilitation programs to treat canal stenosis and dyspareunia. Finally, due to the impact of deconditioning and fatigue following cancer treatment, patients may benefit from education on cardiovascular exercise and activity pacing; posture training; and core muscle strengthening. Treatment plans are individualized based on examination findings and patient goals and typically range from four to twelve sessions.

## Sexual health

### Sexual dysfunction in female survivors

More than half of women with ASCC will endure some form of sexual dysfunction after CRT, including effects on desire, arousal, orgasm and/or pain [35, 92]. A significant number of women who were previously sexually active report becoming inactive following treatment [93–95]. Because chemotherapy targets rapidly dividing cells and is given systemically, it directly targets the ovaries and may lead to decreased estradiol and symptoms of early menopause, which include but are not limited to: vaginal dryness; hot flushes; and decreased libido [93]. Some women will recover from chemotherapy-induced menopause, though this is highly dependent on age, and the existing data is largely derived from breast or gynecologic cancer patients [96, 97].

Additionally, pelvic radiation has short term consequences including inflammation, erythema, and desquamation that can cause significant vaginal pain and dyspareunia. Over time, however, the long-term effects of pelvic radiotherapy will result in decreased oxygenation of the tissue, leading to fibrosis, stenosis and adhesions that can leave the vagina shorter, narrower, and less elastic [92]. Studies have reported up to 79% prevalence of grade 1–3 vaginal stenosis in women after definitive CRT for ASCC, therefore early intervention is imperative [98]. Female patients should be referred to gynecology for early evaluation and discussion of gynecological and sexual health after CRT, ideally with initiation of vaginal dilator therapy within four to 8 weeks after completion of therapy, when indicated.

The mainstays of prevention of sexual health toxicities following treatment for ASCC have focused on vaginal dilator therapy. The premise is that by manually dilating the vagina with a phallic shaped device, women will be able to prevent many of long-term toxicities that lead to vaginal shortening, fibrosis, and stenosis which cause dyspareunia. Unfortunately, despite dilators being promoted in a majority of survivorship programs, the data supporting their use is extremely limited. Indeed, a 2014 Cochrane review found “there is no reliable evidence to show that routine, regular vaginal dilation during radiotherapy treatment prevents stenosis or improves quality of life” [99]. A significant limitation in the majority of vaginal dilator studies is the lack of control group and lack of compliance [100, 101]. Additionally there is no uniform practice or evidence-based guideline for when to initiate dilator therapy or for how often or how long to continue [101]. Further research is necessary to determine who derives benefit from dilator use as well as optimal strategies to prevent radiation toxicity.

While the majority of literature focuses on vaginal dilator therapy to prevent local radiation toxicity, recently there is a new focus on ovarian suppression at the time of treatment to prevent menopause induced by CRT. Despite mixed early data on ovarian suppression during chemotherapy [102], a recent randomized controlled trial of ovarian suppression in breast cancer patients showed that patients who received gonadotropin-releasing hormone agonist had reduced rates of premature menopause without evidence of worsened disease-related outcomes [103]. Additionally, studies are actively investigating multimodal treatments that focus on the psychological effects of treatment, which also have a significant effect on sexual health and can affect the most critical domains of desire and arousal [93]. Any intervention to improve sexual function should ensure that a comprehensive, multidisciplinary approach is employed, focusing on the psychology, endocrinology and physiology of sexual expression and satisfaction and addresses all domains of sexual health.

### Sexual dysfunction in male survivors

Although data on sexual function in male survivors of ASCC is sparse, studies consistently demonstrate that male survivors have significantly impaired sexual function when compared to age and gender-matched controls [33, 34] and even compared to colorectal cancer survivors [16]. Extrapolating from the literature regarding men who have received external radiation therapy for prostate cancer, erectile dysfunction (ED), orgasmic dysfunction, and pain are the most common domains of sexual dysfunction for men following pelvic radiation [104]. Cross-sectional studies show ED likely affects most male ASCC survivors, regardless of age [16, 28, 29, 33, 35].

While it has been hypothesized that sexual dysfunction in males following treatment for ASCC is due to tumor fibrosis and pelvic radiation, a correlation has not been found between radiation dose and sexual dysfunction [35]. ED associated with pelvic radiation may be exacerbated by traditional risk factors for penile arterial insufficiency such as hypertension, hyperlipidemia, cigarette smoking, and diabetes mellitus [105–107]. Psychological distress and relationship strain experienced by ASCC survivors may also contribute to psychogenic ED [108, 109].

Phosphodiesterase type-5 inhibitors (PDE5is) are the general first line approach for treatment of ED, and some data supports the efficacy of PDE5is to treat ED associated with pelvic radiotherapy in cancer survivors [110–112]. PDE5is act as amplifiers of the normal erectile physiology and are dependent on intact libido, sexual stimulation, sensory pathways, and other myriad factors that must be present in normal erectile function.

PDE5i use in patients with cardiovascular disease requires consideration of cardiovascular risk and concurrent medications or substance use (nitrate-containing medications and amyl nitrate inhalers are absolutely contraindicated). Patients with high-risk cardiovascular disease should have their cardiovascular status stabilized prior to resuming sexual activity [113]. Options for second line therapies for ED not responsive to PDE5is include vacuum erectile devices, intracavernosal injections, and transurethral alprostadil. Use of a penile prosthesis may be efficacious as a third-line therapy. However, these indications have been developed in men without cancer, and efficacy in ASCC survivors or recipients of pelvic radiotherapy has not been well studied.

In addition to ED, cross-sectional studies of ASCC survivors note that fecal incontinence is a prominent treatment sequela. For patients who practice anal receptive intercourse (ARI), fecal incontinence might impede sexual function; however, the effect of ASCC treatment on ARI has not been previously studied. Consideration should be given to provide advice regarding safer sex practices, including lubricant usage, and screening for sexually transmitted infections.

## HIV management

Infection with HIV is one of the strongest risk factors for ASCC [114–117]. Fortunately, survival for individuals with HIV on anti-retroviral therapy (ART) is comparable to that of individuals without HIV [118–120]. Treatment is similar to that of the general population, although special attention is needed to evaluate the adequacy of ART and to determine whether additional antimicrobial prophylaxis is indicated.

Current standards dictate that the HIV viral load of patients stable on ART should be monitored at least every 6 months to ensure that it remains undetectable [121]. Whether more frequent testing is needed during or following exposure to chemotherapy is unknown, but an approach that includes monitoring once a month for the first 3 months of chemotherapy and every 3 months thereafter has been proposed [122, 123]. This approach is reasonable given that patients may have suboptimal ART adherence during chemotherapy, drug-drug-interactions may decrease ART effectiveness, and the identification of early virologic failure can prevent complications.

PLWH who are not on ART are at risk for infections associated with depleted cell-mediated immunity. Guidelines recommend antimicrobial prophylaxis against such opportunistic infections based on the number of remaining CD4 positive T-lymphocytes (CD4+ T-cells) in circulation [124]. These guidelines should be followed for PLWH who are ART-naïve and about to initiate chemotherapy, with anticipation of decreases in CD4+ T-cells below the threshold for which prophylaxis related to HIV infection is recommended. It is unclear, however, whether these decreases truly reflect impaired cell-mediated immunity, and hence whether prophylaxis is warranted in patients in whom CD4+ T-cell counts are preserved and viral load are suppressed up until the initiation of CRT. Despite this, monitoring of and initiation of prophylaxis depending on CD4+ T-cell count is recommended by experts, especially since it is reasonably tolerated and easy to administer [122, 123]. Additional prophylaxis recommendations specific to individual chemotherapy regimens are centered around degree and duration of neutropenia; however, this is generally unnecessary with ASCC treatment during which prolonged neutropenia is uncommon.

## Psychosocial wellbeing

NCCN guidelines recommend routine screening to assess the level and nature of emotional distress in all cancer survivors [125]. These guidelines provide guidance about the timing of screening, highlight patient factors associated with distress, and suggest a framework for determining when and to whom to refer emotionally distressed patients [125]. Patients treated with combined CRT for ASCC have a high burden of dysfunction and long-term sequelae [33], consequently levels of anxiety are significantly higher when compared to the general population and health-related quality of life scores show significant impaired in quality of life [33]. Reluctance to disclose sensitive issues may impede optimal management of symptoms; therefore, direct questioning by providers regarding sensitive issues, including bowel function, sexual health, and psychosocial distress, may facilitate communication.

Even in the absence of a clinical event that would trigger distress, many patients will be affected by fear of cancer recurrence, which can persist even when risk of recurrence is low, and is associated with poorer quality of life [126, 127]. At a center which offers psycho-oncology services, we routinely offer referrals for psychotherapy; however, for centers without onsite services, qualified therapist referrals can be found in mental health provider directories [128, 129]. Additionally, the Anal Cancer Foundation is a national non-profit which offers a peer-to-peer support program that can be accessed either online or in-person (Table 3) [131].

**Table 3:** Resources for providers and patients

Psychosocial support	
• Qualified therapist referral: Search United States ZIP code to find local therapist certified by Psychology Today. www.psychologytoday.com/us	
• Peer support: In-person, phone, online connection with another anal cancerpatient. https://www.analcancerfoundation.org/find-support/patient-support/connect-with-a-peer/	
Physical fitness and nutrition	
• Exercise program: YMCA 12-week fitness program specialized for cancer survivors, including those with physical restrictions. Free or reduced cost. https://www.livestrong.org/ymca-search	
• Evidence-based guidelines: The American Cancer Society has put forth guidelines on nutrition and diet for cancer survivors, including literature for clinicians [130] and patients. https://www.cancer.org/health-care-professionals/american-cancer-society-prevention-early-detection-guidelines/nupa-guidelines-for-cancer-survivors.html	
Disease surveillance	
• High-resolution anoscopy: Search providers across the United States that offer high-resolution anoscopy. https://analcancerinfo.ucsf.edu/hra-provider-list	
• Provider trainings in high-resolution anoscopy are offered worldwide by the International Anal Neoplasia Society and include continuing medical education credits. https://ians.wildapricot.org/HRA-Course-Overview	

Because of the unique risk factors and treatments associated with their disease, ASCC patients may perceive social stigma and experience shame. An ASCC survivor must contend with societal views on HPV and on sexual behavior. As providers, we aim to demonstrate sensitivity and immediate responsiveness to any patient expressions of fear of stigma or of shame/guilt.

PLWH and men who have sex with men (MSM) already face stigma, which may be compounded by a diagnosis of ASCC. Patients from sexual and/or gender minorities may be less likely to share information with medical providers because of concerns about stigma or discrimination [132]. As medical providers, we make every effort to address the unique needs of our lesbian, gay, bisexual, transgender, and/or queer (LGBTQ) patients and to minimize health disparities in these populations. Interventions may include seeking education about sexual and social history-taking and taking steps that signal inclusiveness to patients, such as displaying LBGTQ-friendly signs or registering with the Gay and Lesbian Medical Association’s Provider Directory [133]. It is also essential for providers to acknowledge and examine how our own beliefs and biases may influence our care for patients [134, 135].

## Surveillance practices and survivorship care delivery

The short and long-term sequelae of definitive CRT are complex and require a multidisciplinary approach. At our institution, care of ASCC patients after successful completion of treatment is delivered in the context of a multidisciplinary survivorship clinic, with representation of clinicians from oncology, surgery, radiation oncology, anal dysplasia, primary care, infectious diseases, gynecology, nutrition, psycho-oncology, social work, urology, and other disciplines. Transgender individuals may also benefit from endocrinology consultation or specialists in transgender health to optimize safe hormone management. In a complex treatment paradigm requiring multiple health care providers, the oncology survivorship provider serves as a navigator, prioritizing subspecialist referrals and synthesizing recommendations from a variety of disciplines (Table 3).

**Fig. 3:**
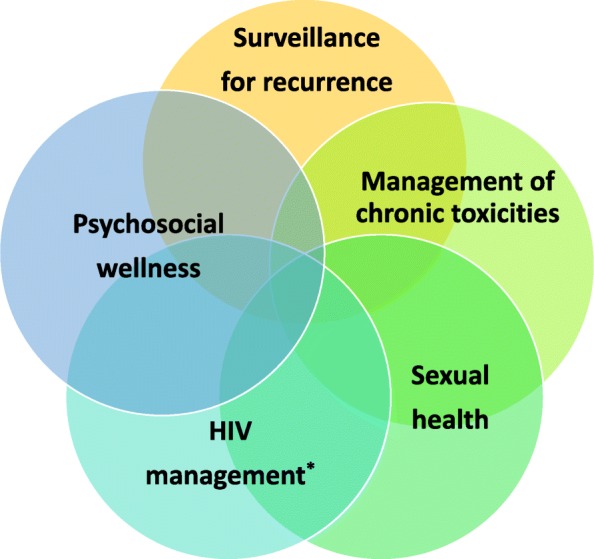
Core domains of a multi-disciplinary model of survivorship care for patients with anal squamous cell carcinoma. *For HIV-infected patients only

The goals of survivorship care for patients with ASCC following completion of definitive CRT are summarized in Fig. 3. A typical encounter in our Survivorship Clinic aims to address the following:

1. Provision of a Survivorship Care Plan to the patient and primary care provider with clearly documented surveillance guidelines [136];
2. Ensuring appropriate surveillance is performed;
3. Evaluation and management of acute and long-term toxicities; and
4. Promotion of risk-reduction strategies for cancer survivors including exercise [137], maintenance of a healthy weight, sleep hygiene, a plant-based diet, and avoidance of salt and refined foods [138]

Patients should be seen at frequent intervals post completion of CRT until resolution of acute side-effects of treatment. Acute toxicities secondary to definitive CRT have been documented to include: myelosuppression (50%), skin toxicity (65%), bowel dysfunction (17%), constipation (3%), fecal incontinence (9%), proctitis (17%), and fatigue (25%) [139]. With resolution of acute toxicities, patients can then transition to clinical evaluations at three monthly intervals for surveillance imaging and screening for long-term toxicities.

Per NCCN guidelines, surveillance should include digital rectal exam and inguinal node palpation every three to 6 months for 5 years, proctoscopy or anoscopy every six to 12 months for 3 years, and CT scans of the chest, abdomen, and pelvis annually for 3years (Table 4) [141]. A PET/CT with contrast performed at 6 months after completion of CRT has been shown to be the best predictor of a complete response to treatment [142] and is our standard institutional practice. Additionally, patients who have undergone pelvic radiation for HPV related cancers are at higher risk for developing subsequent cancers near the radiated sites [143, 144] and remain at higher risk for other HPV-related malignancies, especially cervical, oral, and pharyngeal cancers [145, 146]. Thus, regular gynecologic visits with cervical cytology and dental examinations are recommended, though there is no consensus regarding the frequency and role for HPV testing in this population (Table 4).

**Table 4:** Survivorship care elements and intervals

Months since end of treatment	1–3	3	6	9	12	18	24	30	36	42	48	54	60
Clinic visit with inguinal node palpation and DRE	**X**	**X**	**X**	**X**	**X**	**X**	**X**	**X**	**X**	**X**	**X**	**X**	**X**
Imaging		*PET/CT*	*PET/CT*		**CT CAP**		**CT CAP**		**CT CAP**				
Anoscopy		*X*	**X**	*X*	**X**	*X*	**X**	*X*	**X**				
Gynecology evaluation for women	*X*	*Follow-up interval is determined by findings at baseline evaluation and baseline HPV testing*											
HIV viral load (if applicable)	*Every three months or every six months following stabilization* [140]												

Bold: practices according to National Comprehensive Cancer Network Guidelines (Version 2.2017) [139]

Italics: additional survivorship care delivered at our institution

*DRE* digital rectal exam, *PET* positron emission tomography scan, *CT* computerized tomography, *CAP* computed tomography abdomen and pelvis

The relationship between HPV and ASCC supports the use of HRA as surveillance modality for disease recurrence. The UCSF Anal Neoplasia Clinic employs an alternative screening model in which primary care providers perform HRA and anal cytology screening of our survivor population to evaluate for local recurrence. Most patients are able to undergo surveillance in the clinic, with HRA with biopsies of abnormal lesions performed at three or 4 month intervals (Table 4). HRA capacity is increasing through training workshops offered worldwide through the International Anal Neoplasia Society [147] and other organizations; however, there is still a need to better educate, inform, and train more providers to perform HRA.

Due to the myriad challenges ASCC survivors face, these patients derive significant benefit from a care model that facilitates navigation across a variety of medical specialties. Integration of the primary care provider (PCP) into survivorship care is imperative to ensure that patients resume age-appropriate health care maintenance and to ensure that the PCP is able to assume complete care for the patient. Survivorship care can be transitioned to the PCP during or after the surveillance period is complete.

## Conclusions

In summary, a growing population of ASCC survivors faces a complex burden of physical and psychosocial sequelae. Our approaches to a range of chronic toxicities, sexual health, HIV management in affected patients, psychosocial wellbeing, and surveillance for disease recurrence are reviewed here. This review provides a rigorous and comprehensive summary of the evidence that informs our multi-disciplinary practice, accompanied by expert opinion. In the absence of prospective studies for this rare disease, we believe this review will serve as a resource for oncology care providers, PCP’s, and subspecialists who encounter ASCC survivors in clinical practice. While we acknowledge that every setting may not have the comprehensive services available that has been developed for these patients at our institution, we provide references to link providers to knowledge and resources to facilitate appropriate referrals. Moreover, the emotional and social issues experienced by ASCC survivors may be ameliorated by provider awareness and ability to normalize the experiences of this unique patient group.

Our ongoing research is directed at evaluating the impact of this multi-disciplinary approach in a cohort of ASCC survivors. Future clinical trials and observational studies should collect patient reported outcome measures in addition to acute and chronic toxicities. Recent development of a new, validated quality of life questionnaire specific to anal cancer (EORTC QLQ-ANL27) may facilitate future capture of sub-clinical late effects that cause suffering and functional impairment [148]. In addition, future research should include prospective studies that aim to identify effective medical and behavioral interventions that improve the quality of life for patients following treatment for ASCC.

## Data Availability

Works cited are publicly available.
